# Supplementary material for: Comprehensive characterization of human color discrimination thresholds
Source: eLife. 2026 Jul 31;14:RP108943. doi: 10.7554/eLife.108943 (PMC13427347; doi:10.7554/eLife.108943)
Supplement: Peer review history

# Editor report: eLife Assessment

**DOI:** 10.7554/eLife.108943.3.sa0

**Authors:** Krystel R Huxlin

**Affiliations:** University of Rochester United States

**Keywords:** Compelling, Important

This **important** study describes a novel Bayesian psychophysical approach that efficiently measures how well humans can discriminate between colors across the entire isoluminant plane. The evidence was considered **compelling**, as it included successful model validation against hold-out data and published datasets. This approach could prove to be of use to color vision scientists, as well as to those who employ computational psychophysics and attempt to model perceptual stimulus fields with smooth variations over coordinate spaces.

# Referee report: Reviewer #1 (Public review)

**DOI:** 10.7554/eLife.108943.3.sa1

Summary:

This paper presents an ambitious and technically impressive attempt to map how well humans can discriminate between colours across the entire isoluminant plane. The authors introduce a novel Wishart Process Psychophysical Model (WPPM) - a Bayesian method that estimates how visual noise varies across colour space. Using an adaptive sampling procedure, they then obtain a dense set of discrimination thresholds from relatively few trials, producing a smooth, continuous map of perceptual sensitivity. They validate their procedure by comparing actual and predicted thresholds at an independent set of sample points. The work is a valuable contribution to computational psychophysics and offers a promising framework for modelling other perceptual stimulus fields more generally.

Strengths:

The approach is elegant and well-described, and the data are of high quality. The writing throughout is clear and the figures are clean (elegant in fact) and do a good job of explaining how the analysis was performed. The whole paper is tremendously thorough and the technical appendices and attention to detail are impressive (for example, a huge amount of data about calibration, variability of the stim system over time etc). This should be a touchstone for other papers that use calibrated colour stimuli.

Comments on revised version:

The authors have addressed all the issues I raised to my satisfaction.

# Referee report: Reviewer #3 (Public review)

**DOI:** 10.7554/eLife.108943.3.sa2

Summary:

This study presents a powerful and rigorous approach for characterizing stimulus discriminability throughout a sensory manifold, and is applied to the specific context of predicting color discrimination thresholds across the chromatic plane.

Strengths:

Color discrimination has played a fundamental role in studies of human color vision and for color applications, but as the authors note, remains poorly characterized. The study leverages the assumption that thresholds should vary smoothly and systematically within the space, and validates this with their own tests and comparisons with previous studies.

Comments on revised version:

My comments have been addressed.

# Author comment: Author response

**DOI:** 10.7554/eLife.108943.3.sa3

**Authors:** Fangfang Hong, Ruby Bouhassira, Jason Chow, Craig Sanders, Michael Shvartsman, Phillip Guan, Alex H Williams, David H Brainard

**Affiliations:** University of Pennsylvania Philadelphia United States; University of Pennsylvania Philadelphia United States; Meta Seattle United States; Meta Seattle United States; Meta Seattle United States; Meta Seattle United States; New York University New York United States; University of Pennsylvania Philadelphia United States

The following is the authors’ response to the original reviews.

We would like to thank the editors and the reviewers for the thorough and insightful comments and suggestions. Addressing them has strengthened our manuscript. We have carefully addressed all reviewer comments, as described in detail below, as well as additional comments we received from others. In addition, we made two substantive updates to the manuscript:

(1) We improved the estimation of uncertainty in the model predictions by computing 95% confidence intervals using 120 bootstrapped datasets (instead of the 100% of 10 bootstrapped datasets in the original submission) to match the number of bootstrap for the validation dataset.

(2) We selected a slightly different hyperparameter value based on follow-up analyses suggested by Reviewer 1, which provided very useful information.

Importantly, none of these changes alter the main results or conclusions of the paper.

Beyond these changes and those outlined below, we also worked to improve the clarity of the prose throughout as well as added various additional citations to the literature.

**Public Reviews:**

**Reviewer #1 (Public review):**

Summary:

This paper presents an ambitious and technically impressive attempt to map how well humans can discriminate between colours across the entire isoluminant plane. The authors introduce a novel Wishart Process Psychophysical Model (WPPM) - a Bayesian method that estimates how visual noise varies across colour space. Using an adaptive sampling procedure, they then obtain a dense set of discrimination thresholds from relatively few trials, producing a smooth, continuous map of perceptual sensitivity. They validate their procedure by comparing actual and predicted thresholds at an independent set of sample points. The work is a valuable contribution to computational psychophysics and offers a promising framework for modelling other perceptual stimulus fields more generally.

Strengths:

The approach is elegant and well-described (I learned a lot!), and the data are of high quality. The writing throughout is clear, and the figures are clean (elegant in fact) and do a good job of explaining how the analysis was performed. The whole paper is tremendously thorough, and the technical appendices and attention to detail are impressive (for example, a huge amount of data about calibration, variability of the stim system over time, etc). This should be a touchstone for other papers that use calibrated colour stimuli.

Weaknesses:

Overall, the paper works as a general validation of the WPPM approach. Importantly, the authors validate the model for the particular stimuli that they use by testing model predictions against novel sample locations that were not part of the fitting procedure (Figure 2). The agreement is pretty good, and there is no overall bias (perhaps local bias?), but they do note a statistically-significant deviation in the shape of the threshold ellipses. The data also deviate significantly from historical measurements, and I think the paper would be considerably stronger with additional analyses to test the generality of its conclusions and to make clearer how they connect with classical colour vision research. In particular, three points could use some extra work:

(1) Smoothness prior.

The WPPM assumes that perceptual noise changes smoothly across colour space, but the degree of smoothness (the eta parameter) must affect the results. I did not see an analysis of its effects - it seems to be fixed at 0.5 (line 650). The authors claim that because the confidence intervals of the MOCS and the model thresholds overlap (line 223), the smoothing is not a problem, but this might just be because the thresholds are noisy. A systematic analysis varying this parameter (or at least testing a few other values), and reporting both predictive accuracy and anisotropy magnitude, would clarify whether the model's smoothness assumption is permitting or suppressing genuine structure in the data. Is the gamma parameter also similarly important? In particular, does changing the underlying smoothness constraint alter the systematic deviation between the model and the MOCS thresholds? The authors have thought about this (of course! - line 224), but also note a discrepancy (line 238). I also wonder if it would be possible to do some analysis on the posterior, which might also show if there are some regions of color space where this matters more than others? The reason for doing this is, in part, motivated by the third point below - it's not clear how well the fits here agree with historical data.

Thank you for raising this important point. We have now added analyses of the effects of the two smoothness-related hyperparameters, ε and γ (see Appendix 10).

First, we swept a range of values for each hyperparameter (ε: 0.1 – 1; γ: 0.000001 – 0.003) and evaluated model performance using 5-fold cross-validation of the dataset used to fit the WPPM, quantifying predictive accuracy on held-out test data. We used the mean negative log likelihood averaged across the held-out data in the cross validation as our measure of predictive accuracy (Figs. S27-31).

The two hyperparameters affect cross-validation accuracy in a similar manner. With γ fixed at 0.0003, predictive accuracy is highest for ε in the range of approximately 0.3–0.5 and drops quite rapidly for ε < 0.3. We attribute this drop to oversmoothing. Cross-validation accuracy also decreases, albeit more gradually, for ε > 0.5. We attribute this to increased variance due to undersmoothing relative to the power of our datasets. Similarly, with ε fixed at 0.4, predictive accuracy is highest for γ values between approximately 0.0001 and 0.001, declines rapidly for smaller γ (oversmoothing), and more slowly for larger γ (undersmoothing).

Second, we examined how the hyperparameter ε affected the agreement between the WPPM fit and the MOCS validation data. Specifically, at each ε, for each participant, we computed the linear regression between WPPM thresholds and validation thresholds at 25 reference locations. Then, we examined the slope and correlation coefficient of all participants as a function of ε. We found a classic bias–variance tradeoff. Excessive smoothness introduces bias by failing to capture structure in the data, whereas insufficient smoothness increases variance in model predictions. These results further support a choice of ε = 0.4 as lying near the optimal balance between bias and variance (Fig. S32).

Based on these analyses, we selected for the final analysis ε = 0.4, slightly smaller than the preregistered value used in the original submission (0.5), while retaining the original value of γ (0.0003).

We now discuss these reasons for changing this value in the revision, as well as provide a more general discussion of the importance and practicalities of hyperparameter choice in Bayesian approaches to analyzing data (Discussion / Prior specification).

(2) Comparison with simpler models. It would help to see whether the full WPPM is genuinely required. Clearly, the data (both here and from historical papers) require some sort of anisotropy in the fitting - the sensitivities decrease as the stimuli move away from the adaptation point. But it's >not< clear how much the fits benefit from the full parameterisation used here. Perhaps fits for a small hierarchy of simpler models - starting with isotropic Gaussian noise (as a sort of 'null baseline') and progressing to a few low-dimensional variants - would reveal how much predictive power is gained by adding spatially varying anisotropy. This would demonstrate that the model's complexity is justified by the data.

In the 5-fold cross-validation analysis described above (and now presented in Appendix 10), we found that when ε or γ is small, the stronger smoothness constraint leads to threshold ellipses that are nearly identical to each other across color space. Under these conditions, model predictions show poor accuracy on held-out test data and lead to poor predictions of the validation data. This observation addresses the underlying point raised by the reviewer, albeit in a different way than suggested: it shows that a degree of spatially varying anisotropy is necessary to capture the structure of the data. We now make this point in the paper (Discussion / Prior specification).

More broadly, we employed the WPPM as a prior that imposed smoothness but not much other obvious structure, and used this to learn about the psychometric field. We are currently working to understand how we can best use our current data to improve the prior we would apply to future measurements. There are a number of approaches to this. One would be to seek a parametric mechanistic model that can describe the current data, and to the extent this is possible formulate prior distributions over the parameters of the model. The results reported here thus provide a foundation for deriving and evaluating more structured priors that would even more efficiently leverage future datasets, but with the feature that they impose more structure. We have added this perspective to the Discussion / Extensions of the WPPM framework.

(3) Quantitative comparison to historical data. The paper currently compares its results to MacAdam, Krauskopf & Karl, and Danilova & Mollon only by visual inspection. It is hard to extract and scale actual data from historical papers, but from the quality of the plotting here, it looks like the authors have achieved this, and so quantitative comparisons are possible. The MacAdam data comparisons are pretty interesting - in particular, the orientations of the long axes of the threshold ellipses do not really seem to line up between the two datasets - and I thought that the orientation of those ellipses was a critical feature of the MacAdam data. Quantitative comparisons (perhaps overall correlations, which should be immune to scaling issues, axis-ratio, orientation, or RMS differences) would give concrete measures of the quality of the model. I know the authors spend a lot of time comparing to the CIE data, and this is great.... But re-expressing the fitted thresholds in CIE or DKL coordinates, and comparing them directly with classical datasets, would make the paper's claims of "agreement" much more convincing.

Although we are sympathetic to this request, we have chosen not to implement the sort of quantitative comparison requested by the reviewer. The reason is that an important feature of color thresholds is that they depend on the spatial (e.g. Kelly, 1974; Poirson & Wandell, 1996; Danilova & Mollon, 2025) and temporal (e.g. Kelly, 1974) properties of the stimuli, and on the observer’s state of adaptation (e.g. Loomis & Berger, 1979; Krauskopf & Gegenfurtner, 1992). Because (as the reviewer notes below) the spatial and temporal properties of our stimuli were not matched to those of the comparison datasets, our purpose in making these comparisons was to examine qualitative agreement, as well as to situate our results in the literature and to demonstrate that our approach allows us to read out thresholds around the references and in the color spaces used in other studies. We would not expect detailed quantitative agreement with the current dataset because of differences in stimuli.

As a consequence of this, we think we would be overreaching to quantify the differences between our data and classic datasets. This consideration is particularly important for the MacAdam measurements, where because of the matching adjustment procedure used, the observer’s state of adaptation is likely to have varied (by amounts that are difficult to estimate) from one reference to the next (e.g. Danilova & Mollon, 2025). We have clarified the manuscript with respect to these points (Results / Comparison with previous measurements).

A point to make on this topic is that an important and interesting future direction that emerges from our work is to develop efficient methods to characterize the dependence of the full discrimination field on ancillary variables, such as those that describe spatial and temporal properties and/or the state of adaptation, which we now also mention in the paper (Discussion / Implications for the mechanisms of color perception). Although not the primary motivation, doing so would enable comparison of data with a wider range of studies.

We do agree that the comparisons to CIELAB predictions work better when we express them in CIELAB, and have now done so (Fig. 3D; Fig. S24-S26).

Kelly, D. H. (1974). "Spatio-temporal frequency characteristics of color-vision mechanisms." Journal of the Optical Society of America 64(7): 983–990.

Poirson, A. B. and B. A. Wandell (1996). "Pattern-color separable pathways predict sensitivity to simple colored patterns " Vision Research 36(4): 515–526.

Danilova, M. V. and J. D. Mollon (2025). "Effect of stimulus size on chromatic discrimination." Journal of the Optical Society of America A 42(5).

Loomis, J. M. and T. Berger (1979). "Effects of chromatic adaptation on color discrimination and color appearance." Vision Research 19(8): 891–901.

Krauskopf, J., Gegenfurtner, K. (1992). "Color discrimination and adaptation." Vision Research 32(11): 2165–2175.

Overall, this is a creative and technically sophisticated paper that will be of broad interest to vision scientists. It is probably already a definitive method paper showing how we can sample sensitivity accurately across colour space (and other visual stimulus spaces). But I think that until the comparison with historical datasets is made clear (and, for example, how the optimal smoothness parameters are estimated), it has slightly less to tell us about human colour vision. This might actually be fine - perhaps we just need the methods?

Related to this, I'd also note that the authors chose a very non-standard stimulus to perform these measurements with (a rendered 3D 'Greebley' blob). This does have the advantage of some sort of ecological validity. But it has the significant disadvantage that it is unlike all the other (much simpler) stimuli that have been used in the past - and this is likely to be one of the reasons why the current (fitted) data do not seem to sit in very good agreement with historical measurements.

As the reviewer notes, our stimuli head in the direction of ecological validity (see also Hedjar et al., 2025) and indeed this was a consideration when we chose them, at the cost of limiting the degree of comparison we can make with prior studies (as discussed above). Another reason we chose our stimuli is that they enable the current data to be used as a basis of comparison with stimuli where we add specularity, change object shape, and vary object pose in the future. These manipulations are not possible with flat matte patches. Such experiments are of interest to us, as they will tell us about how effectively color may be used to differentiate stimuli in cases where other ecologically important variables co-vary. We now mention this motivation in the paper (Results / Task and Stimuli).

Hedjar, L., M. Toscani and K. R. Gegenfurtner (2025). "Importance of hue: color discrimination of three-dimensional objects and two-dimensional discs." Journal of the Optical Society of America A 42(5).

**Reviewer #2 (Public review):**

Summary:

Hong et al. present a new method that uses a Wishart process to dramatically increase the efficiency of measuring visual sensitivity as a function of stimulus parameters for stimuli that vary in a multidimensional space. Importantly, they have validated their model against their own hold-out data and against 3 published datasets, as well as against colour spaces aimed at 'perceptual uniformity' by equating JNDs. Their model achieves high predictive success and could be usefully applied in colour vision science and psychophysics more generally, and to tackle analogous problems in neuroscience featuring smooth variation over coordinate spaces.

Strengths:

(1) This research makes a substantial contribution by providing a new method to very significantly increase the efficiency with which inferences about visual sensitivity can be drawn, so much so that it will open up new research avenues that were previously not feasible. Secondly, the methods are well thought out and unusually robust. The authors made a lot of effort to validate their model, but also to put their results in the context of existing results on colour discrimination, transforming their results to present them in the same colour spaces as used by previous authors to allow direct comparisons. Hold-out validation is a great way to test the model, and this has been done for an unusually large number of observers (by the standards of colour discrimination research). Thirdly, they make their code and materials freely available with the intention of supporting progress and innovation. These tools are likely to be widely used in vision science, and could of course be used to address analogous problems for other sensory modalities and beyond.

Weaknesses:

It would be nice to better understand what constraints the choice of basis functions puts on the space of possible solutions. More generally, could there be particular features of colour discrimination (e.g., rapid changes near the white point) that the model captures less well.

This comment bears conceptual similarity to Reviewer 1’s question about the hyperparameters of our prior, as it is basically asking whether we might be oversmoothing through the choice of form and number of basis functions. The hyperparameter sweeps we now present suggest that within the choice of basis functions we used, we are operating at a reasonable point on the bias-variance tradeoff curve - we can see bias emerging with a smoother prior, and variance increasing with a less smooth prior. Our expectation is that varying the smoothness of the prior in other ways, such as by varying the form and number of the basis functions, would lead to similar tradeoffs.

We did perform one additional check that shows, within our current framework, that adding more basis functions is unlikely to change things much. This was to plot the fit weights as a function of Chebyshev basis order (Figure S4 in Appendix 2). These decline to near zero at the highest order we used, suggesting that adding more would not alter the inferred psychometric field, given our hyperparameter choices. Although we could explore this question further by explicitly fitting the data using more basis functions along with different hyperparameter choices, or different functional forms for the basis functions, we decided not to pursue this in favor of performing the other additional analyses we now present.

We resonate with the reviewer’s concern that assuming smoothness, both by assuming that isoperformance contours are elliptical and by assuming that these vary smoothly with reference, might cause us to miss features of the true underlying field in cases where that field varies rapidly or the isoperformance contours are asymmetric or non-elliptical. Our approach to this was to measure the validation thresholds and demonstrate that any bias in our WPPM-inferred field is small for these measurements. Because we shared the reviewer’s intuition that the adapting point is a candidate location where there might be less smooth variation, we measured a validation threshold at this reference for every subject. Nonetheless, we only measured in one direction around the adapting reference for each subject. We considered validation approaches where we measured full ellipses at a set of validation references, but we were worried about effects of uncertainty reduction and perceptual learning which might distort thresholds at highly sampled locations.

It is the case that if one wanted to study the discrimination field in more detail around a particular reference, one could concentrate trials in a smaller model space around that reference, and for the same number of trials use a prior with less smoothness relative to the underlying stimulus space. Indeed, simply halving the size of the stimulus space that maps onto the [-1,1] model space and keeping the same prior over the model space effectively halves the degree of smoothness expressed with respect to the stimulus space. Thus our methods could prove useful in studying more rapid variations in the discrimination field if one hypothesized that they might occur around particular reference choices, but this would still rest upon the elliptical assumption. To relax that assumption, one could use the threshold field estimation methods implemented in AEPsych, which incorporate a smoothness assumption but do not assume elliptical isoperformance contours. Weakening the prior in this way would, however, increase trial demand to obtain similar measurement precision.

As a general matter, we don’t think it is possible to leverage smoothness for trial efficiency on the one hand and at the same time be completely sure that there isn’t some aspect to the underlying ground truth that has been smoothed over. Carefully choosing the degree of prior smoothness together with the number of experimental trials in the context of a particular content problem is an important part of bringing the WPPM and related methods to bear, and one where simulation and held-out data both play an important role.

We now bring these points out more fully in the paper (Discussion / Extensions of the WPPM framework; Discussion / Prior specification).

Chen, C.-C., J. M. Foley and D. H. Brainard (2000). "Detection of chromoluminance patterns on chromoluminance pedestals I: threshold measurements." Vision Research 40(7): 773–788.

The substantial individual differences evident in Figure S20 (comparison with Krauskopf and Gegenfurtner, 1992) are interesting in this context. Some observers show radial biases for the discrimination ellipses away from the white point, some show biases along the negative diagonal (with major axes oriented parallel to the blue-yellow axis), and others show a mixture of the two biases. Are these genuine individual differences, or could the model be performing less accurately in this desaturated region of colour space?

We agree that these differences are interesting. We have now added more complete bootstrapped confidence regions in these (Appendix 8) and the other comparison figures (Appendix 6, 7, 9), so that an estimate of measurement precision is directly available in these figures. These confidence regions suggest that the individual differences in this region of color space are real. A longer-term goal is to develop more mechanistic models that can account for individual subject data through parameter choice. This might lead to insight into what differs in the visual system across individuals.

**Reviewer #3 (Public review):**

Summary:

This study presents a powerful and rigorous approach for characterizing stimulus discriminability throughout a sensory manifold, and is applied to the specific context of predicting color discrimination thresholds across the chromatic plane.

Strengths:

Color discrimination has played a fundamental role in studies of human color vision and for color applications, but as the authors note, it remains poorly characterized. The study leverages the assumption that thresholds should vary smoothly and systematically within the space, and validates this with their own tests and comparisons with previous studies.

Weaknesses:

The paper assumes that threshold variations are due to changes in the level of intrinsic noise at different stimulus levels. However, it's not clear to me why they could not also be explained by nonlinearities in the responses, with fixed noise. Indeed, most accounts of contrast coding (which the study is at least in part measuring because the presentation kept the adapt point close to the gray background chromaticity, and thus measured increment thresholds), assume a nonlinear contrast response function, which can at least as easily explain why the thresholds were higher for colors farther from the gray point. It would be very helpful if a section could be added that explains why noise differences rather than signal differences are assumed and how these could be distinguished. If they cannot, then it would be better to allow for both and refer to the variation in terms of S/N rather than N alone.

We agree with the reviewer. We are measuring SNR and attributing it to noise, but cannot identify from the data whether changes in SNR across color spaces are due to changes in noise, to a nonlinear relationship between stimulus space and the observer’s response space with noise in the response space held fixed, or both. We now make this point where we introduce the Results / Wishart Process Psychophysical Model and reiterate it in the Discussion / Extensions of the

WPPM framework.

Related to this point, the authors note that the thresholds should depend on a number of additional factors, including the spatial and temporal properties and the state of adaptation. However, many of these again seem to be more likely to affect the signal than the noise.

We don’t disagree. Indeed, as we noted in our response to a comment by Reviewer 1 and above in the context of individual differences, we are very interested in developing a mechanistically plausible model that accounts for the data. If we or others are able to do so, that would provide a basis for parsing performance into separate signal and noise effects. And if such a model has natural ways in which additional variables affect its predictions, measuring the effects of these variables would be a way to provide evidence in favor of the model (Discussion / Implication for the mechanisms of color perception - Extensions of the WPPM framework).

An advantage of the approach is that it makes no assumptions about the underlying mechanisms. However, the choice to sample only within the equiluminant plane is itself a mechanistic assumption, and these could potentially be leveraged for deciding how to sample to improve the characterization and efficiency. For example, given what we know about early color coding, would it be more (or less) efficient to select samples based on a DKL space, etc?

The more we are willing to assume about the structure of the psychometric field, the more efficiently we can measure it. As the reviewer correctly notes, this principle applies to trial placement as well. We are currently using an adaptive method (AEPsych) that starts with a fairly weak smoothness prior and attempts to place trials using heuristics that aim to minimize the expected uncertainty in the posterior. As we learn more about the discrimination field, we should be able to leverage stronger priors to increase trial efficiency. This point is closely related to one we made above about developing stronger priors that capture what we have learned in this study. Such priors could also help improve trial placement. For a prior that has a relatively small number of parameters, for example, perhaps a mechanistic prior, methods such as Quest+ (Watson, 2017) may be used for trial placement.

Watson, A. B. (2017). "QUEST+: A general multidimensional Bayesian adaptive psychometric method." J Vis 17(3): 10.

**Recommendations for the authors:**

**Reviewer #1 (Recommendations for the authors):**

I do not think that the authors need to perform additional experiments. However, I would like to see some additional analyses regarding the assumptions made in the fitting procedure and how they affect the final maps.

I also think some more quantitative comparisons with historical data would be valuable - at the moment, a lot of the comparisons are simply 'by eye'.

It would have been nice to have the code and data available during the review procedure - I'm sure these will be released with excellent documentation?

We addressed the first two points in the public review section. The code is now available online as is the data. These links are now provided in the paper (Methods and Materials / Data and code availability).

**Reviewer #2 (Recommendations for the authors):**

Minor points

I have a few suggestions for additions and small changes.

(1) Several examples of covariance matrix fields are shown in Figure 1, 4, but these are for simulated examples. It would be nice to see the fields actually fit the data! I would be interested in seeing this for all participants in an Appendix, and maybe for participant CH in the main paper?

We have made the changes (see Figure 4 and Figure S3).

(2) I have not worked through all the math in the appendices line by line, but it seems to be complete, and the model validation results speak for themselves. I think the authors have done a pretty good job of explaining the model conceptually (not easy), but I struggled with the 'weighted sum' step in Figure 4 and the main text. I would appreciate a bit more hand-holding here, e.g, why is an 'overcomplete' representation needed as an intermediate, and providing an intuition of why there are 12 matrices in the overcomplete representation and what each matrix in this representation represents.

We have now added more explanations in the figure legend and text (Fig. 4 and Methods and Materials / The Wishart Process Psychometric Model).

(3) Individual differences: There is a section on this in the manuscript, and it's concluded that there are only "modest" individual differences. However, in Figure S20, the individual differences, I think, are huge and place observers almost in qualitatively different categories! Some observers show a radial bias in discrimination ellipses, others seem to show basically a bias along the negative diagonal, and others a mixture of both biases. These ellipses are at a desaturated part of colour space - is it possible that there are some rapid changes in the underlying noise in this region that the Wishart fit has not captured due to relatively sparse sampling or the fact that the basis functions are all fairly low spatial frequency? I wondered whether the results are constrained by the choice of Cartesian rather than polar basis functions, e.g, polar basis functions may have better allowed fine-grained changes near the white point but slower changes at higher saturations away from the white point.

We agree that the individual differences are meaningful and, in some cases, quite pronounced. Our intent in describing the differences as “modest” was to emphasize that the overall structure of the psychometric fields remains broadly consistent across observers. We have revised the Results to note and more fully describe these differences.

Regarding the possibility that sharp changes in the underlying noise near the achromatic point might not be fully captured by the current model, we agree that this is an important consideration. The current implementation uses relatively low-order Chebyshev basis functions that primarily capture smooth global variations in the psychometric field. While validation analyses indicate that these basis functions capture the dominant structure in the data, they may be less sensitive to sharp local variations such as those that could occur near the white point. Future work could address this by mapping the model space to a smaller region around the achromatic reference or by exploring alternative basis sets (e.g., polar or Zernike functions) that may better capture such localized structure. This is discussed above in this response and now addressed in Discussion / Extensions of the WPPM framework.

On sampling, I wondered if the results might have been biased by the strongly biased ellipse that occurs at the grey point. If not, and the model is accurate in this region of colour space, I think this figure does show some large individual differences, and it would be good to comment on these in the individual differences section of the manuscript.

Based on our analysis of trial placement (Fig. S1), the adaptive algorithm does not appear to have disproportionately concentrated trials near the gray point. In fact, more trials were allocated to the edges of the stimulus space than to the center. This suggests that the WPPM estimates are unlikely to be driven primarily by performance in the gray region. In addition, we examined the threshold ellipses around the gray reference in DKL space and found that they are broadly consistent across participants (Figs. S22–S23). Together, these analyses suggest that the anisotropy observed near the gray point reflects a genuine property of the psychometric field rather than an artifact of the sampling procedure.

As noted just above, we have added additional text about individual differences in the Results and referenced it in the Discussion.

(4) The manuscript seems unusually free of typographical errors, but I noticed that in many places "Krauskopf and Karl 1992" is cited! Also, I think something has gone wrong with the legend to Figure 2 - perhaps the order of panels was swapped around, but the legend was not fully updated. There is a repeated reference to the "summary of regression slopes" which seems to be in 2 positions, after C and G. It would make more sense to label panel G as D and progress from there, or switch the order of the panels so that G is on the bottom row.

Thank you for catching those errors. They are now fixed.

**Reviewer #3 (Recommendations for the authors):**

A minor point (or perhaps major if your last name is Gegenfurtner) is that the reference to Krauskopf and Karl is incorrect.

They are now fixed.
